# Mathematical Model for Localised and Surface Heat Flux of the Human Body Obtained from Measurements Performed with a Calorimetry Minisensor

**DOI:** 10.3390/s17122749

**Published:** 2017-11-28

**Authors:** Fabiola Socorro, Pedro Jesús Rodríguez de Rivera, Miriam Rodríguez de Rivera, Manuel Rodríguez de Rivera

**Affiliations:** Departamento de Física, Universidad de Las Palmas de Gran Canaria, E-35017 Las Palmas de Gran Canaria, Spain; fabiola.socorro@ulpgc.es (F.S.); pedrojrdrs@gmail.com (P.J.R.d.R.); miriam.mrdrs@gmail.com (Mi.R.d.R.)

**Keywords:** direct calorimetry, heat conduction calorimeters, isothermal calorimeters, medical calorimetry, non-differential calorimeters

## Abstract

The accuracy of the direct and local measurements of the heat power dissipated by the surface of the human body, using a calorimetry minisensor, is directly related to the calibration rigor of the sensor and the correct interpretation of the experimental results. For this, it is necessary to know the characteristics of the body’s local heat dissipation. When the sensor is placed on the surface of the human body, the body reacts until a steady state is reached. We propose a mathematical model that represents the rate of heat flow at a given location on the surface of a human body by the sum of a series of exponentials: *W*(*t*) = *A*_0_ + *∑A_i_*exp(*−t*/*τ_i_*). In this way, transient and steady states of heat dissipation can be interpreted. This hypothesis has been tested by simulating the operation of the sensor. At the steady state, the power detected in the measurement area (4 cm^2^) varies depending on the sensor’s thermostat temperature, as well as the physical state of the subject. For instance, for a thermostat temperature of 24 °C, this power can vary between 100–250 mW in a healthy adult. In the transient state, two exponentials are sufficient to represent this dissipation, with 3 and 70 s being the mean values of its time constants.

## 1. Introduction

In the study of physico-chemical or biological processes, the energy developed in the process has always been of great interest. It can be measured using calorimetry instruments, which have primarily been developed in the field of thermal analysis [1]. The uncertainty of energy and/or heat power measurements is relatively high due to three main factors: external disturbances, lack of prior knowledge of the process under study, and an inability to precisely replicate the process. For example, these instruments are calibrated using Joule dissipations and additional common processes that serve as references [2]. However, the new process under examination differs greatly to these procedures. Therefore, the uncertainty of energy and/or heat power measurements may increase by up to 10% depending on the particular case [3,4].

In calorimetry, the process under study takes place in an enclosed area of measurement so as to reproduce as accurately as possible the physico-chemical or biological process under study. In many cases, in order to reduce the uncertainty of the measurement, it is necessary to manufacture specific instruments for each case under study. This has led to the development of a myriad of thermal analysis instruments [5].

In our present study, our aim is to perform a local measurement of the surface heat dissipated from the human body. It is a direct measurement of a continuous energy process that will be altered by placing a sensor on the surface of the human body. In this particular calorimetry application, it is not possible to completely isolate the process under study and thus, this measurement does not comply with normal calorimetry standards [6]. For this application, two calorimetric sensors have been constructed. They perform under the same operating procedure but are of different sizes; the first prototype has a detection area measuring 6 × 6 cm^2^ [7,8,9,10], while the second prototype has a 2 × 2 cm^2^ detection area [11].

Several works explaining the operating principle and the measurement technique of these sensors have been published. In these works, the main method of measuring power involves determining the mean power generated during the time of application of the sensor on the surface of the human body. The observation of the numerous measurements made with both sensors [7,8,9,10,11] shows different calorimetric curves, demonstrating a characteristic model of heat dissipation over time. The main objective of this work is to study this temporal relationship. The proposed model correctly represents the rate of heat flow from a human body by the sum of a series of exponentials: *W*(*t*) *= A*_0_ *+ ∑A_i_*exp(*−t*/*τ_i_*). In this way, the transient and steady states of this dissipation can be interpreted. This hypothesis is verified by simulating the sensor, treating it as a linear and invariant multiple-input multiple-output (MIMO) system. We emphasize that the assumption of the shape of the input signal (heat flow from a human body) simplifies the deconvolutive procedure.

With the emergence of digital infrared cameras, a considerable amount of research has been undertaken into examining the measurement of the body surface temperature [12]. In some cases, this research is conducted in order to study the interaction between human thermophysiology and the external environment [13]. In other cases, the local temperature can be used to monitor and detect inflammation associated with knee replacements [14,15], rheumatoid arthritis [16], osteoarthritis [17], allergies [18], frozen shoulders [19] and tendinitis [20]. Thus, measurements made with the calorimetry minisensor can become a valuable complement to pathological studies.

The main objective of this work is the accurate determination of the surface and localized heat power dissipated from the human body. Therefore, we have built a second calorimetry minisensor, which is similar to the first minisensor, in order to verify that the thermal results obtained are of the same order of magnitude. In the development of this work, we will provide a brief description of the instrument, which will include the operating methods for calibration and for the measurements on the human body. Eventually, we will present the results and conclusions, from which we can highlight that the numerical results provided by the sensor are objective and very useful for the study of the heat dissipated from the human body.

## 2. Materials and Methods

### 2.1. Calorimetry Minisensor

The calorimetry minisensor consists of a thermopile located between the measurement area (2 × 2 cm^2^) and a thermostat programmed at a constant temperature. The thermopile produced a calorimetric signal thanks to the Seebeck effect. The thermostat comprised a small (10 × 12 × 3 mm^3^) aluminium block containing a heating resistor and an RTD sensor. The thermostat also includes a cooling system based around a thermopile, which absorbed heat from the thermostat through the Peltier effect, and an aluminium heat sink (with its corresponding fan) attached to the hot surface of the cooling thermopile. The heat flow that passes across the first thermopile is determined from the equations that define the minisensor’s operations. The minisensor used in this work has already been described in detail in a previous work [11].

**Figure 1:**
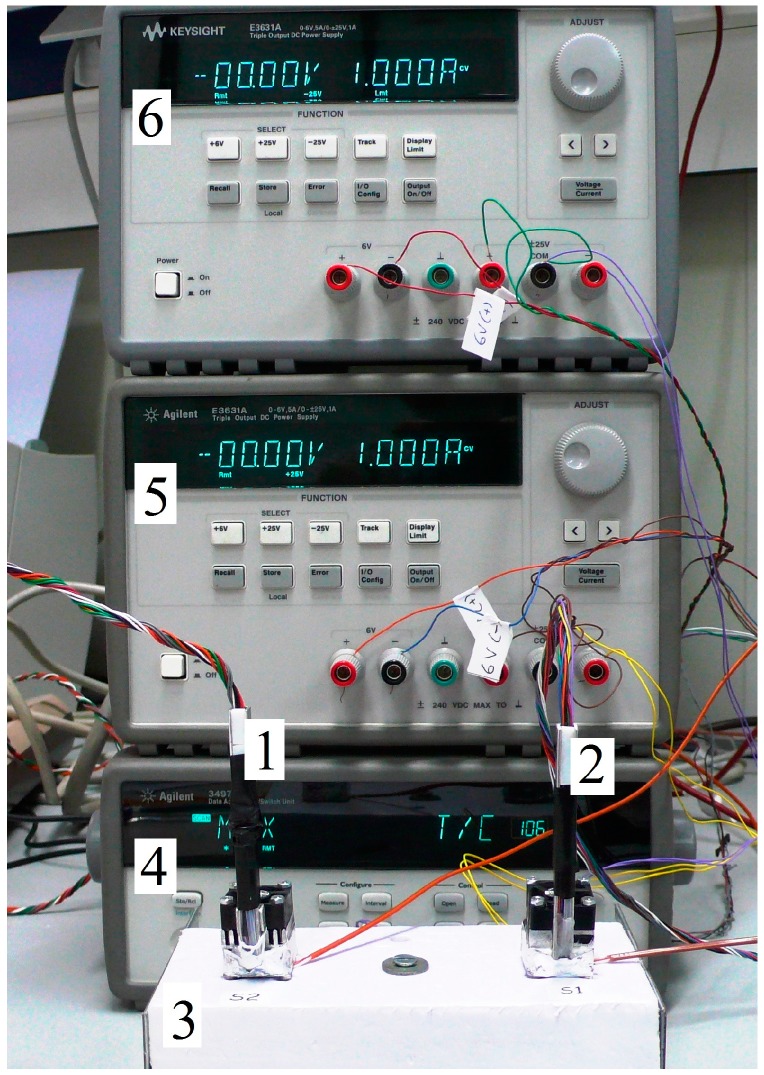
Minisensors (**1**,**2**) on its calibration base (**3**), data acquisition system (**4**) and the two power supplies (**5**,**6**).

We have built a second minisensor to obtain simultaneous measurements on two different points of the body in order to validate the heat power measurements of the initial minisensor, and to determine the thermal resistance of each sensor. This second minisensor is similar to the first, but differs enough to require a specific calibration. Whilst the data system acquisition is sufficient for both minisensors, the measuring equipment requires a second programmable power supply (Figure 1). The control and data acquisition program has been adapted for both minisensors, but this version of the program has been implemented in C++ to ensure the consistency of the sampling period. The sampling period used in all the measurements was 1 s. This is the highest possible frequency, as the thermostat temperature is measured with a 1 mK resolution with four wires and using Pt-100 sensors. The multimeter used (Agilent 34970A, Keysight, Santa Rosa, CA, USA) has been programmed to measure with a resolution of 0.1 mΩ, and this requires sampling periods of no less than 1 s.

### 2.2. Calibration

The second minisensor requires calibration, while the first requires recalibration since the fastening screws have been replaced by new ones. These new screws allow the adaptation of the minisensor to the new calibration base that magnetically holds the minisensors, making it easier to manipulate. Any modification made to the instrument requires recalibration as the change in the thermal capacity and conductivity of the materials alters the sensitivity and time constants of the minisensor.

The calibration of these instruments requires the prior consideration of an operating model, which has already been described in detail in the previous work [11]. As a summary, the model treats the instrument as a linear time invariant system with two inputs and two outputs. The inputs are (1) the power *W*_1_(*t*) dissipated in the resistor placed on the calibration base or the power dissipated by the human body, which passes across the sensor in the direction of the thermostat (in positive or negative sense, depending on the thermostat temperature) and (2) the power *W*_2_(*t*) dissipated in the thermostat in order to maintain its temperature constant. The outcomes are (1) the calorimetric signal *y*_1_(*t*) provided by the measurement thermopile and (2) the thermostat temperature *y*_2_(*t*). In this way, we can define four transfer functions (*TF_i_*) of this multiple-input multiple-output (MIMO) system that describe the relationship between the inputs and outputs in the Laplace domain as follows:

(1) $$\left(\begin{array}{cc} TF_{1} & TF_{2}\\ TF_{3} & TF_{4} \end{array}\right)\left(\begin{array}{c} \Delta W_{1}(s)\\ \Delta W_{2}(s) \end{array}\right)=\left(\begin{array}{c} \Delta Y_{1}(s)\\ \Delta Y_{2}(s) \end{array}\right)$$

The four *TF_i_* have the same poles, but different sensitivities and zeros [9,11]. Given the signal-to-noise ratio of the signals, this system can be well identified with two poles and one zero for each *TF_i_*:

(2) $$TF_{i}(s)=K_{i}\frac{\left(1+s\tau_{i}^{*}\right)}{\left(1+s\tau_{1})(1+s\tau_{2}\right)}$$

where *K_i_* is the sensitivity or steady state response to a unit step, while *τ_i_ = −*1/*s_i_* and τ_i_* = −1/s_i_* (*s_i_* represents the poles and s_i_* the zeros for each *TF_i_*). 

**Figure 2:**
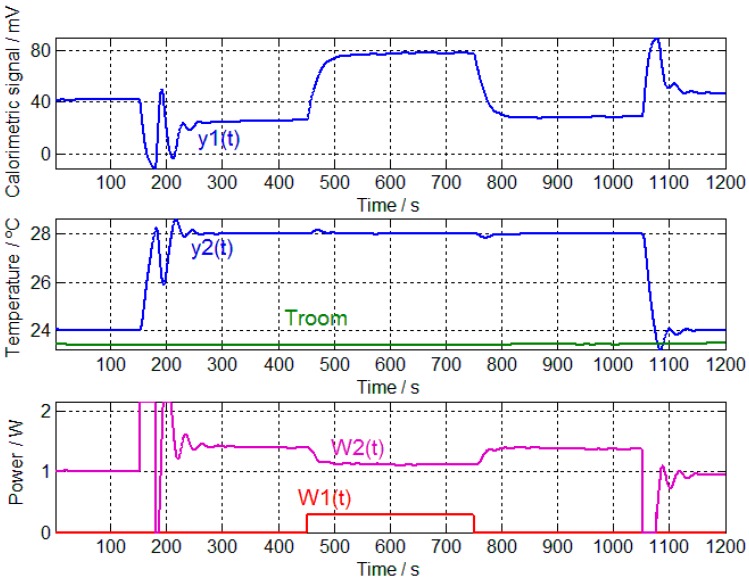
Calibration measurements, including calorimetric signal *y*_1_(*t*), thermostat temperature *y*_2_(*t*), room temperature *T_room_*, power dissipated in the resistor placed on the calibration base *W*_1_(*t*) and power dissipated in the thermostat *W*_2_(*t*).

**Figure 3:**
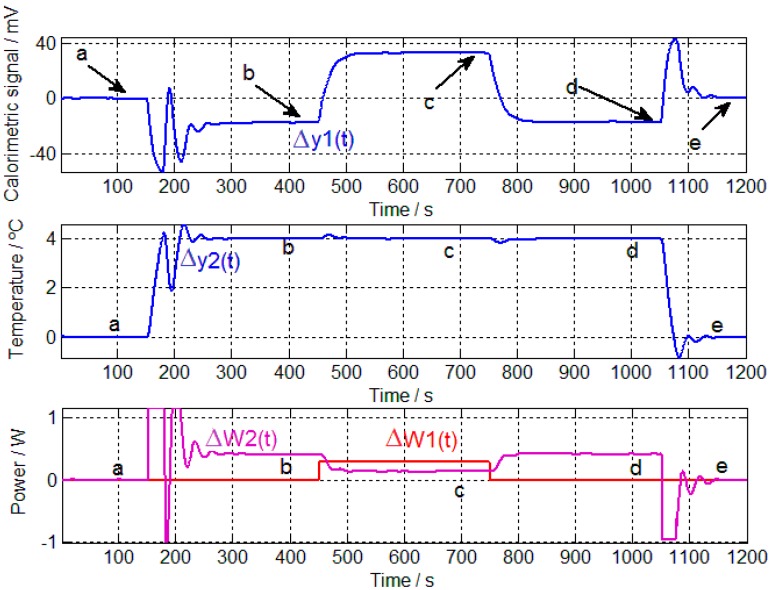
Calibration measurements after correcting the baselines, including calorimetric signal ∆*y*_1_(*t*), thermostat temperature ∆*y*_2_(*t*), power dissipated in the resistor placed on the calibration base ∆*W*_1_(*t*) and power dissipated in the thermostat ∆*W*_2_(*t*). The five steady states are indicated (**a**–**e**).

For the calibration, the following procedure was executed. First, the sensitivities of the four transfer functions are determined, allowing for calculation of the poles and zeros of each *TF_i_*. A calibration measurement is shown in Figure 2, which requires the thermostat temperature to be maintained at 24 °C (from *t* = 0 to *t* = 150 s), before being changed to 28 °C (from *t* = 151 s to *t* = 1050 s) and finally returning to the initial temperature of 24 °C (from *t* = 1051 s to the end). When the thermostat reaches the steady state of 28 °C, 300 mW is dissipated in the resistor, which is placed on the calibration base for 5 min (from *t* = 451 to *t* = 750 s). The sensitivities are determined based on signal values obtained in the steady state. Baseline signals must be corrected (Figure 3) before the system equations can be applied for each steady state (Zones a–e of Figure 3):

(3) $$\begin{array}{l} K_{1}\Delta W_{1}+K_{2}\Delta W_{2}=\Delta y_{1}\\ K_{3}\Delta W_{1}+K_{4}\Delta W_{2}=\Delta y_{2} \end{array}$$

At each steady state, the oscillations of the curves used for calibration are ±0.2 mV for *y*_1_(*t*); ±5 mK for *y*_2_(*t*); ±0.1 mW for *W*_1_(*t*); and ±10 mW for *W*_2_(*t*).

Poles and zeros (*s_i_* and s*_i_) or the inverse of their opposites (*τ_i_* and τ*_I_ of Equation (2)) were determined by minimizing a certain error criterion between the experimental (∆*y_exp_*) and theoretical curves calculated (∆*y_cal_*) with equations from the model (Equation (2)). This was achieved using Nelder–Mead simplex search algorithm [21] and MatLab software [22]. The error criterion selected was the mean squared error given by the following equation:

(4) $$\sigma_{y}=\frac{1}{N}\sqrt{\sum_{i=1}^{N}{\left(\Delta y_{\exp}[i]-\Delta y_{cal}[i]\right)}^{2}}$$

The results of the calibration (Table 1) show that although the sensors are the same, they have slight differences in their construction. Hence, each sensor requires a specific calibration.

### 2.3. Measurement Method

**Figure 4:**
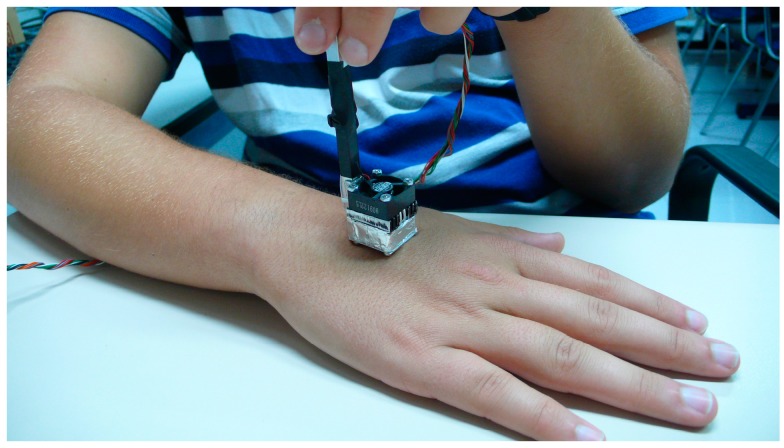
Calorimetry minisensor on the right hand of the subject.

Calibration measurements are all made with the minisensor located on its calibration base (Figure 1). However, a basic measurement in the human body for a programmed thermostat temperature has three phases: (1) the minisensor is placed on the calibration base until the set temperature reaches the steady state (initial baseline); (2) the minisensor is placed on the surface of the human body for the required time (1–5 min, (Figure 4)); and finally, (3) the minisensor is returned to the base until the signals return to their initial baseline. Generally, each phase takes 5 min, resulting in a total of 15 min for a set temperature.

**Figure 5:**
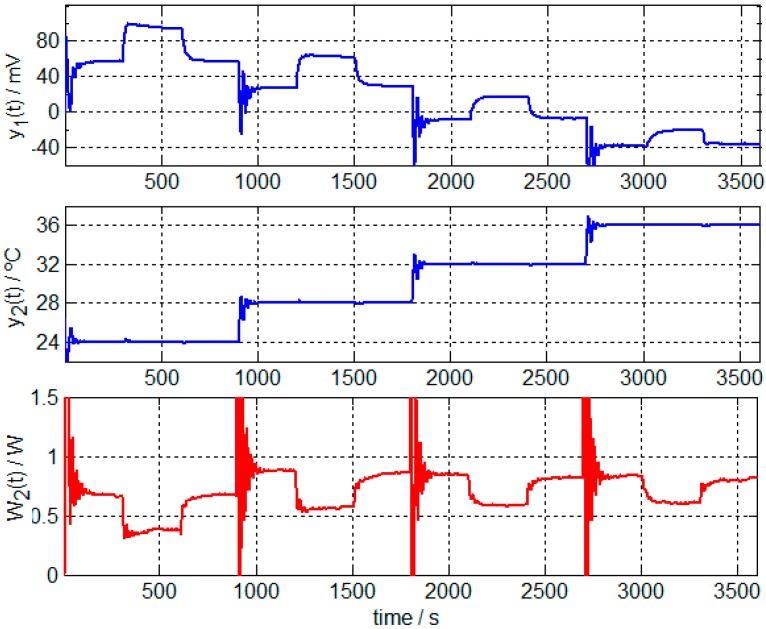
Curves corresponding to four consecutive measurements on the human body (right hand of a healthy 23-year-old male subject) for four different temperatures of the thermostat. These measurements include calorimetric signal *y*_1_(*t*), thermostat temperature *y*_2_(*t*) and power dissipated in the thermostat *W*_2_(*t*).

Figure 5 displays the curves corresponding to four consecutive measurements in which the thermostat was set to 24, 28, 32 and 36 °C. In this case, the measurements were made on the right hand of a healthy 23-year-old male subject. In order to maintain these temperatures and not to saturate the minisensor, the cooling thermopile must be powered with an appropriate voltage. Saturation of the sensor occurs when the power dissipated in the heating resistor located in the thermostat reaches the maximum (upper saturation) or the minimum (lower saturation) values. The determination of the voltage supply of the cooling thermopile requires the *K*_3_ and *K*_4_ sensitivities obtained in the calibration (Table 1). The calculation of this voltage is obtained from the measurement program itself from the equations determined in the previous work [11].

### 2.4. Mathematical Model of the Surface Heat Dissipation of the Human Body

In previous works [9,11], we have considered the power dissipated from the human body as the mean value of the power dissipated during the sensor application period. This hypothesis is correct if the power dissipated from the human body is of the “Heaviside signal” type. If not, it has the disadvantage of depending on the application time. The measurement analysis shows that the transient state of the signals depends not only on the minisensor’s time constants but also on the transient response of the human body.

To represent the heat flux dissipated from the human body in this particular situation, we have considered a mathematical model that assumes that the power passing across the sensor is equal to the sum of a series of exponentials:

(5) $$W_{1}(t)=A_{0}+\sum A_{i}\exp(-t/\tau_{i})$$

The coefficients and the time constants of this power *W*_1_(*t*) are determined by the same method of minimization used to identify the *TF_i_* parameters of the sensor. After this, *TF*_1_ and *TF*_2_ are known (Table 2), while *A*_0_, *Ai* and *τ_i_* are unknown values that form the signal *W*_1_(*t*). This signal *W*_1_(*t*), together with the known *W*_2_(*t*) curve, allows the determination of the calorimetric response *y*_1_(*t*) using the following equation in the temporal space:

(6) $$\tau_{1}\tau_{2}\frac{d^{2}y_{1}(t)}{dt^{2}}+(\tau_{1}+\tau_{2})\frac{dy_{1}(t)}{dt}+y_{1}(t)=K_{1}\left(\tau_{1}^{*}\frac{dW_{1}(t)}{dt}+W_{1}(t)\right)+K_{2}\left(\tau_{2}^{*}\frac{dW_{2}(t)}{dt}+W_{2}(t)\right)$$

Once the curve *y*_1_(*t*) is determined, the chosen error criteria are calculated as follows:

(7) $$\sigma_{y1}=100\sqrt{\sum_{i=1}^{N}{\left(y_{1}{}_{\exp}[i]-y_{1cal}[i]\right)}^{2}/\sum_{i=1}^{N}{\left(y_{1\exp}[i]\right)}^{2}}$$

**Figure 6:**
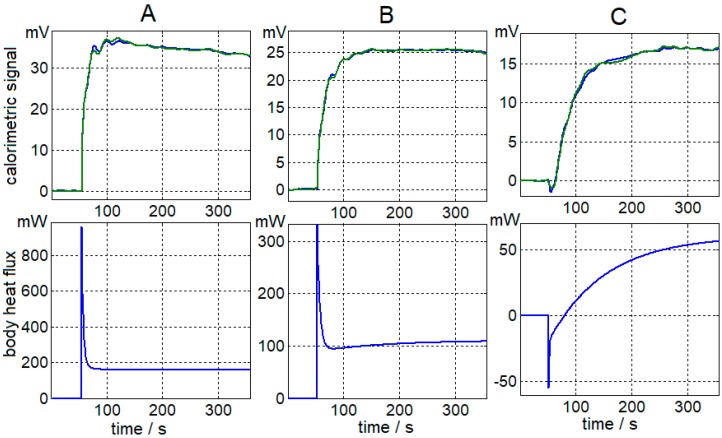
Calorimetric signal adjustment (experimental curve in blue and calculated curve in green). Results of the body heat flux (right hand of a healthy 23-year-old male subject) for three different thermostat temperatures: (**A**) 28 °C, (**B**) 32 °C and (**C**) 36 °C. Figure 5 represents the complete measurements.

Before starting the calculation, the baselines of the curves *y*_1_(*t*) and *W*_2_(*t*) must be corrected. In the calculation process, we have verified that two time constants are sufficient to represent *W*_1_(*t*) and reconstruct *y*_1_(*t*). Figure 6 shows the adjustments of the measurements performed on the right hand of a healthy 23-year-old male subject at the thermostat temperatures of 28, 32 and 36 °C (Figure 5). Table 2 shows the results of the calculation for this case, which are the amplitude values and the time constants of the power (Equation (5)) dissipated from the human body for the different thermostat temperatures.

In addition to the different amplitude of the signals, there is an obvious difference between the three curves shown in Figure 6. In the first curve (Figure 6A), there is an initial positive oscillation, whilst the third curve (Figure 6C) demonstrated that there is a slight negative oscillation. This difference is caused by the difference in thermostat temperature (*Tcal*) and the initial surface temperature of the body (*Tbody* ≈ 32 °C). In the first measurement, *Tcal < Tbody* (*Tcal* = 28 °C); in the second one, *Tcal ≈ Tbody* (*Tcal* = 32 °C); and in the third measurement *Tcal > Tbody* (*Tcal* = 36 °C). These differences are mainly evident in the value of the coefficient *A*_1_. The oscillations observed at the beginning of the calorimetric curve and the power *W*_2_(*t*) (Figure 5 and Figure 6) are similar and are the system’s transient response to the proportional-integral-derivative (PID) temperature control of the thermostat. A greater oscillation indicates a greater temperature difference between the thermostat and the body.

The coefficient *A*_0_ represents the steady-state power and, as expected, its value is different from that obtained as the mean value (*W_mean_*). The results presented in Table 2 show that *A*_0_ *< W_mean_* when *Tcal* < *Tbody_initial_*; *A*_0_ *≈ W_mean_* when *Tcal* ≈ *Tbody_initial_*; and *A*_0_ *> W_mean_* when *Tcal* > *Tbody_initial_*.

## 3. Results

### 3.1. Surface Heat Dissipation of the Human Body

Numerous measurements have been performed on different parts of the human body (hand, wrist, chest and front) for different temperatures of the sensor’s thermostat. Measurements were made on a normally dressed subject, who remained in a resting state (seated position). The functions of the human body are complex and the heat dissipation varies significantly depending on the physical state of the subject. To focus mainly on the sensor’s operation, two series of representative measurements are presented. These measurements were performed on the hand because it is one of the regions of interest for the thermal measurement of the body surface [23] and also for ease of measurement.

The first series of measurements was performed on the right and left hands using the two minisensors. Each series consists of four measurements for four thermostat temperatures. These series are the same as the one shown in Figure 5. The measurements are obtained simultaneously from both hands using the two minisensors. We have performed six series of measurements to create a total of 48 measurements, with 24 on the right hand and 24 on the left hand. Half are made with the first minisensor and the other half with the second minisensor. The measurements were taken on two consecutive days. Before and after each measurement, blood pressure and heart rate were measured, with normal values obtained. The subject is a healthy 23-year-old male. The mean temperature of the room was 24.7 °C and air flow was prevented.

**Figure 7:**
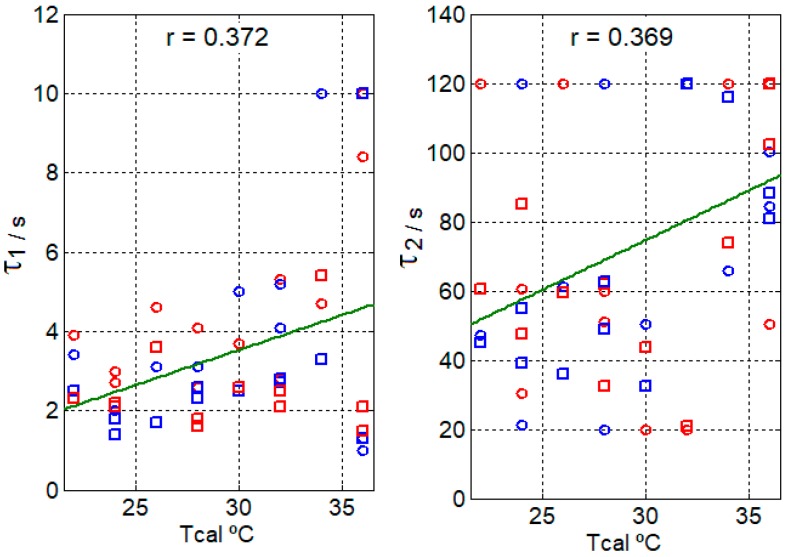
Time constants of the heat flux (Equation (5)) of the right hand (blue points) and the left hand (red points) of a healthy 23-year-old male subject, measured with the minisensor S1 (round) and the minisensor S2 (squares) for different thermostat temperatures (*Tcal*). *r* is Pearson’s linear correlation coefficient.

**Figure 8:**
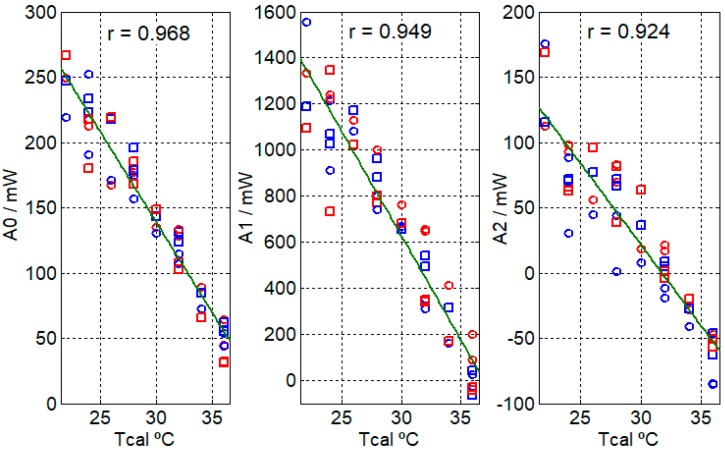
Coefficients *A*_0_, *A*_1_ and *A*_2_ of the heat flux (Equation (5)) of the right hand (blue points) and the left hand (red points) of a healthy 23-year-old male subject, measured with the minisensor S1 (round) and the minisensor S2 (squares) for different thermostat temperatures (*Tcal*). *r* is Pearson’s correlation coefficient.

Per the procedure described in the previous section, the amplitudes and time constants of the heat flux (Equation (5)) have been determined. Figure 7 and Figure 8 display the results for each hand, each sensor and each thermostat temperature (*Tcal*). From these results, we deduce that the heat dissipation of each hand is similar and that there are no significant differences in the measurements made with each minisensor.

**Figure 9:**
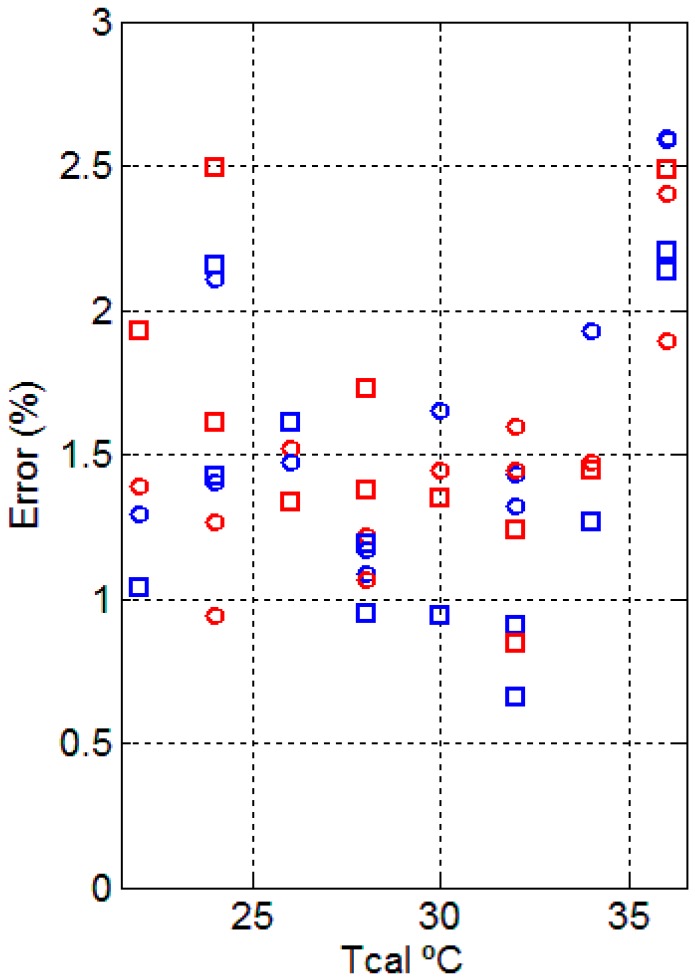
Adjustment errors of the calorimetric curve (Equation (7)) in the determination of the coefficients *A*_0_, *A*_1_ and *A*_2_ represented in Figure 8. Measurements were made on the right hand (blue points) and the left hand (red points) of a healthy 23-year-old male subject, which was measured with the minisensor S1 (round) and the minisensor S2 (squares) for different thermostat temperatures (*Tcal*).

The dispersion of the obtained values for the time constants (Figure 7) is large (Pearson’s linear correlation coefficient (r) is 0.372 for *τ*_1_ and 0.369 for *τ*_2_) due to low frequency oscillations, thus we can consider a mean value of the time constants, the first being 3 s and the second being 70 s. By setting these time constants, the associated amplitudes are recalculated. The resulting errors in the adjustment are considered acceptable since they are less than 3% (Figure 9). It is important to set these time constants so that the values of the amplitudes are clearly independent and the adjusted value of the independent term *A*_0_ is not affected by the second exponential. The amplitudes of *A*_0_, *A*_1_ and *A*_2_ have a clear linear relationship with the thermostat temperature (see Figure 8), with the parameters of the corresponding fitting lines shown in Table 3.

**Figure 10:**
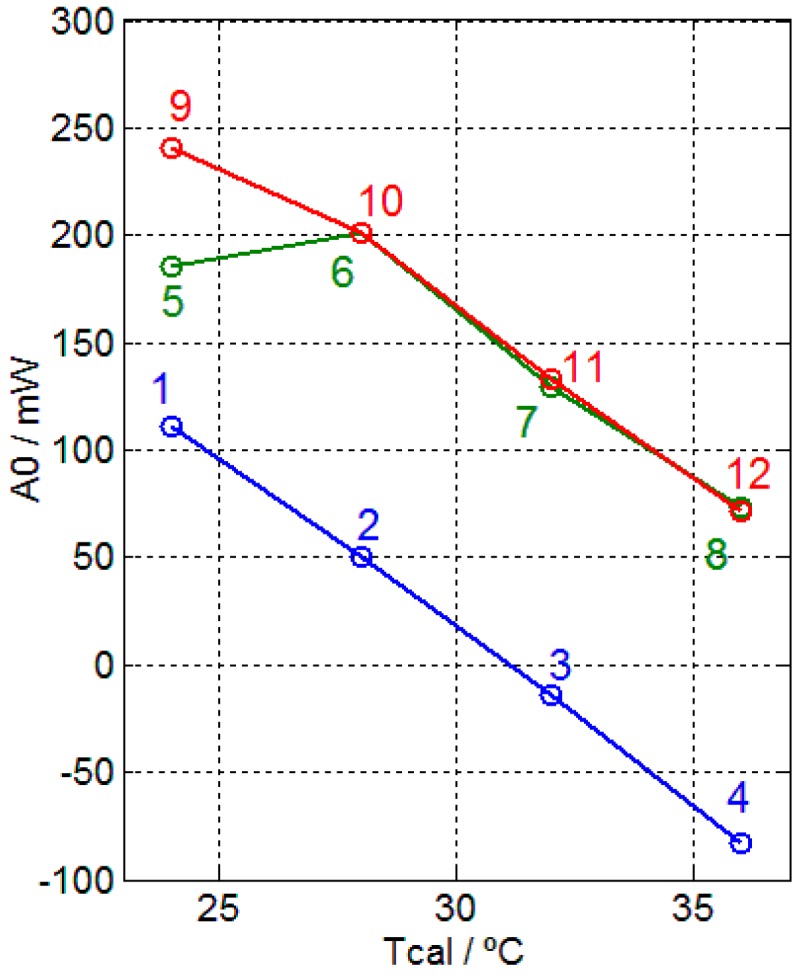
Coefficient *A*_0_ of the heat flux (Equation (5)) of the human body (left hand of a healthy 59-year-old male subject) for different thermostat temperatures (*Tcal*). Measurements made on the same day: 1–4 (morning), 5–12 (afternoon).

In order to study the variations of heat dissipation in different physiological situations of the subject, a second series of representative measurements is shown. This series has been performed on the left hand of a healthy 59-year-old male subject, who remained in a resting state (seated position). Heart rate and blood pressure were also measured before and after each measurement, with normal values obtained. The sequence of measurements is identical to that shown in Figure 5. The first series was made in the morning whilst the subject was cold, while the second and third series were made in the afternoon of the same day, when the subject was not cold. Figure 10 shows the value of the coefficient *A*_0_, which represents the heat flux at the steady state. The order of measurements is marked sequentially: 1–4, 5–8 and 9–12. We can observe that the measured heat flux seems to be related to the subject’s state, which requires further investigation. The transition of line 1–4 to line 9–12 shows the heating of the subject. The measurement 5–8 is a transient measurement between these two steady states (morning and afternoon). The slope of the lines 1–4 and 10–12 is similar; −16.2 mW/K in the first case, −16.1 mW/K in the second case.

### 3.2. Thermal Resistance of the Sensor and the Human Body

**Figure 11:**
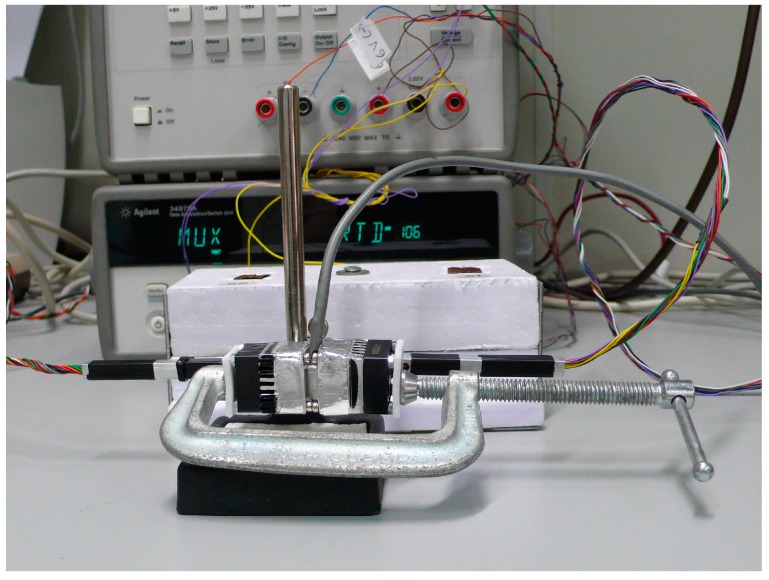
Placement of the two minisensors to determine their thermal resistances.

**Figure 12:**
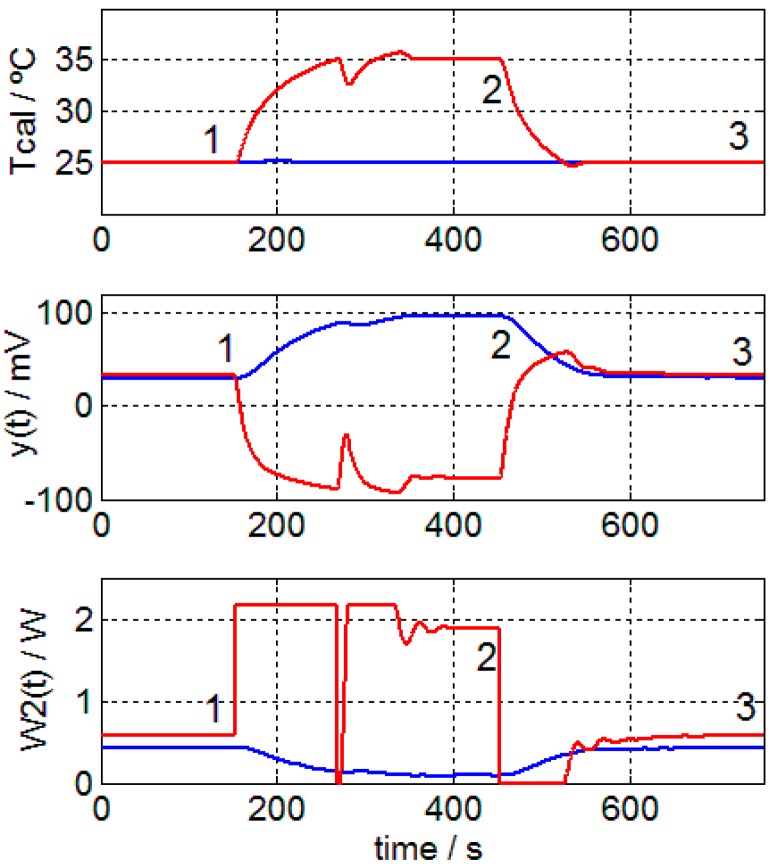
Measurements to determine the thermal resistance of each sensor, including thermostat temperature (*Tcal*), calorimetric signal (*y*(*t*)) and power dissipated in the thermostat (*W*_2_(*t*)). These are curves of the minisensor S1 (blue) and minisensor S2 (red). Steady states are marked 1, 2 and 3.

The linear relationship of *A*_0_ (heat flux at the steady state) with the thermostat temperature suggests that the sensor is capable of providing an order of magnitude of the thermal conductivity of the human body. To test this hypothesis, we should first determine the thermal resistance of each sensor. To do this, the two sensors are brought into contact as shown in Figure 11. An aluminum plate with a temperature sensor inside is placed in the contact zone. The same thermostat temperature is programmed for both sensors (*Tcal*_1_ = *Tcal*_2_ = 25 °C). When the steady state is reached, one minisensor maintains its temperature while the other is subjected to a temperature change that is previously programmed (*Tcal*_1_ = 25 °C and *Tcal*_2_ = 35 °C). Finally, both sensors are returned to the initial temperature (*Tcal*_1_ = *Tcal*_2_ = 25 °C). The steady states of the experimental curves (situations 1–3 of Figure 12) allow the determination of the heat flow from one sensor to another and thus, the total thermal resistance of the set. The heat power obtained is 420 mW, while the thermal resistance of each sensor is *R_sensor_* ≈ 5/0.420 ≈ 12 K/W. Although the measurement is not very precise, as the contact zone between the minisensors should be isolated, it is sufficient to obtain an approximate value of the sensor’s thermal resistance.

The measurement of power on the surface of the human body at several thermostat temperatures (*Tcal*) was determined with the described method. The variation of *A*_0_ with a change in temperature *Tcal* provides the slope of the fitting line, which is directly related to the thermal resistance between two supposed points of different temperatures: the thermostat of the minisensor and the interior of the human body. At the steady state, the heat flux between both points is *A*_0_. Hence, the inverse of the slope of the line is the total thermal resistance (*R_T_*) between both points. The resistance corresponding to the area of the human body will be *R_body_ = R_T_* − *R_sensor_*. 

To determine the thermal conductivity of the human body, we assume for the first approximation that the internal resistance of the human body in the measurement area (*R_body_*) is composed of a flat wall of *S* = 4 cm^2^ of surface, *L* = 1 cm deep and a constant thermal conductivity of *λ*.

(8) $$R_{body}=\frac{1}{\lambda }\frac{L}{S}$$

We assume *L* = 1 cm based on the width of the subjects’ hands (3–3.5 cm). This hypothesis assumes that the dissipation of the human body occurs at a single point and that the internal temperature varies linearly with distance. Obviously, these hypotheses yield incorrect results since the thermal dissipation of the human body is more complex than the proposed mechanism. We must take into account that different parts of the body differ in heat dissipation and/or absorption. We should also consider blood flow, as it plays a role in the maintenance of the body temperature. However, these hypotheses allow us to obtain an order of magnitude of the thermal conductivity so as to compare it with values obtained in literature. Table 4 shows that the results obtained are in the same order of magnitude as the references, which propose values of 0.2–0.7 Wm^−1^K^−1^ [24,25].

## 4. Discussion

In order for the calorimetric minisensor to be of medical utility, the results provided must be consistent and accurate. The main objective of this work is the study of the heat power dissipated by the surface of the human body. A second minisensor has been built to support the first one, and ensure the validity of results. We have used both minisensors simultaneously with changes in the measurement site. Thus, we have confirmed that the results of both minisensors are in the same order of magnitude (Figure 7, Figure 8 and Figure 9).

The proposed model (Equation (5)) for the power dissipated by the surface of the human body acceptably explains the behavior of the body in the presence of the sensor. The independent term *A*_0_ represents the power dissipated by the human body in the steady state. Additionally, the exponentials *A*_1_exp(*−t*/*τ*_1_) and *A*_2_exp(*−t*/*τ*_2_) explain the transient state of the dissipation. The first exponential, which has a time constant of 3 s, represents a signal of the “Dirac pulse” type. This signal is produced due to the discontinuity suffered by the sensor when it passes from the calibration base to the surface of the human body, which is at a different temperature. The second exponential has a mean time constant of 70 s and this heat power is directly related to the human body’s ability to adapt to contact with the minisensor. The values of the amplitudes obtained using the time constants of 3 and 70 s are an acceptable adjustment of the calorimetric curve calculated from the equation that simulates the sensor’s operation (Equation (6)). In all cases, the adjustment error is less than 3% (Figure 9).

Consecutive series of measurements have been performed for different thermostat temperatures in the range of 22–36 °C. We emphasize the importance of performing this type of measurement series for two reasons. First, we can validate all measurements by comparing the linear relationship between temperature and *A*_0_. Secondly, the slope of the line provides an approximate value of the thermal conductivity of the subject’s body, which must be in the normal range. Furthermore, the slope of the fitting line for *A*_2_ must be similar to that of *A*_0_ since these two terms (*A*_0_ and *A*_2_exp(*−t*/*τ*_2_)) are directly associated with the behavior of the human body.

In this work, we obtained several thermal measurements to show the order of magnitude of the surface power dissipated by the human body in the case of contact measurement. In the current configuration of the sensor, the measurement uncertainty is ±10 mW, which has been determined by identical and consecutive measurements from the human body [11]. We have obtained numerous measurements from the body that provide very different values of heat power according to the particular time as well as the physical state of the measured subject. The complex functions of the human body account for the possible measurement uncertainty at the beginning of the analysis of results (Figure 10). In other words, while the surface and interior body temperature is constant in a healthy subject, the heat dissipation involved in maintaining this constant temperature varies widely. 

In summary, the numerical results provided by the sensor are objective and very useful for the study of human physiology. Nevertheless, the relationship between the results and the body’s thermal conductivity requires further investigation.

## Figures and Tables

**Table 1:** Results of the calibration of each minisensor (Equations (1) and (2) parameters) and mean squared errors of the adjustment (Equation (4)).

	S1 Sensor	S2 Sensor
*K*_1_	117.98 ± 0.33 mVW^−1^	132.40 ± 0.48 mVW^−1^
*K*_2_	−51.33 ± 0.50 mVW^−1^	−42.22 ± 0.75 mVW^−1^
*K*_3_	8.98 ± 0.21 KW^−1^	8.67 ± 0.20 KW^−1^
*K*_4_	11.92 ± 0.18 KW^−1^	9.82 ± 0.15 KW^−1^
*τ*_1_	80.8 s	84.0 s
*τ*_2_	9.5 s	8.2 s
*τ*_1_*	64.4 s	73.5 s
*τ*_2_*	108.2 s	96.1 s
*τ*_3_*	0.0 s	0.0 s
*τ*_4_*	22.2 s	18.4 s
*σ*_y1_	0.076 mV	0.066 mV
*σ*_y2_	1.94 mK	2.69 mK
*N*	1200 points	1200 points

**Table 2:** Coefficients and time constants of the heat flux (Equation (5)) of the human body (right hand of a healthy 23-year-old male subject) for four thermostat temperatures (*Tcal*).

*Tcal*/°C	*A*_0_/mW	*A*_1_/mW	*A*_2_/mW	*τ*_1_/s	*τ*_2_/s	Error Equation (7) (%)	*W_mean_*/mW
24	193.7	1156.4	101.0	2.0	21.2	1.29	207.3
28	156.6	779.3	25.4	3.1	20.0	1.03	163.3
32	110.4	244.6	−21.6	5.2	120.0	0.78	103.3
36	60.0	−35.6	−79.0	1.0	100.2	0.60	28.9

**Table 3:** Parameters of the fitting lines (*A_i_* = *α + βT_cal_*) of the coefficients *A*_0_, *A*_1_ and *A*_2_, as represented in Figure 8.

	*α* mW	*β* mW °C^−1^	Error *σ* (Equation (4)) mW	Max. Deviation mW
*A*_0_	556.21	−13.91	2.38	41.86
*A*_1_	3342.53	−90.49	20.06	437.88
*A*_2_	393.95	−12.41	3.43	65.26

**Table 4:** Thermal resistance and thermal conductivity of the human body with subject 1 being a 23-year-old male and subject 2 being a 59-year-old male.

Measurement	Subject	Slope of *A*_0_ mWK^−1^	*R_T_* KW^−1^	*R_body_* KW^−1^	Thermal Conductivity Wm^−1^K^−1^
Table 2	1	−11.2	89.3	77.3	0.32
Table 3	1	−13.9	71.9	59.4	0.42
Figure 8	2	−16.2	61.7	49.3	0.50
Figure 8	2	−16.1	62.1	50.1	0.50
